# Risk Factors for the First Episode of Peritonitis in Southern Chinese Continuous Ambulatory Peritoneal Dialysis Patients

**DOI:** 10.1371/journal.pone.0107485

**Published:** 2014-09-15

**Authors:** Xiaoguang Fan, Rong Huang, Juan Wang, Hongjian Ye, Qunying Guo, Chunyan Yi, Jianxiong Lin, Qian Zhou, Fengmin Shao, Xueqing Yu, Xiao Yang

**Affiliations:** 1 Department of Nephrology, The First Affiliated Hospital, Sun Yat-sen University, Guangzhou, People’s Republic of China; 2 Key Laboratory of Nephrology, Ministry of Health, Guangzhou, People’s Republic of China; 3 Department of Nephrology, Zhengzhou University People's Hospital, Zhengzhou, People’s Republic of China; University of Utah School of Medicine, United States of America

## Abstract

**Background:**

The first episode of peritonitis affects survival of the peritoneal membrane as a medium for dialysis as well as survival of patients. The aim of this study is to investigate risk factors associated with the first episode of peritonitis in Southern Chinese continuous ambulatory peritoneal dialysis (CAPD) patients.

**Methods:**

This is a single-center, retrospective, cohort study. All incident CAPD patients from 1 January 2006 to 31 December 2010 were recruited, and followed up until their first episode of peritonitis or 31 December, 2012. Baseline demographic, socioeconomic, clinical and laboratory data were collected. Cox proportional model was used to determine the factors associated with the first episode of peritonitis.

**Results:**

In a cumulative 30756.5 patient-months follow-up (the median vintage 26.1 months) of 1117 CAPD patients, 309(27.7%) patients presented the first episodes of peritonitis. The cumulative peritonitis-free survival was 86.2%, 78.1%, 71.4% and 57.8% at 1, 2, 3 and 5 year, respectively. The multivariate analysis showed that factors associated with risk for the first episode of peritonitis were elderly patients (>65 years) [hazard ratio (HR) = 1.427, 95% confidence interval (CI) = 1.051 to 1.938, P = 0.023], male(HR = 1.315, 95% CI = 1.028 to 1.684, P = 0.030), lower education level (HR = 1.446, 95% CI: 1.127 to 1.855, P = 0.004) and albumin <38g/L (HR = 1.425, 95% CI: 1.112 to 1.825, P = 0.005).

**Conclusions:**

Older age, male, lower educational level and hypoalbuminemia at the commencement of PD were the risk factors associated with the first episode of peritonitis in Southern Chinese CAPD patients.

## Introduction

Peritonitis is one of the most common and severe complications associated with peritoneal dialysis (PD), constituting the primary cause of catheter loss and exit from the dialysis technique [1]. It has reported that the first episode of peritonitis affects survival of peritoneal membrane as a medium for dialysis as well as survival of the PD patients [2]. A study identified that the time interval until the first episode of peritonitis was shorter in PD patients coming from hemodialysis (HD) as compared to those with no previous experience on renal replacement therapy (RRT) [3]. Another study confirmed the aboriginal and obese PD patients have a higher rate of peritonitis and a shorter time to the first episode of peritonitis [4]. Moreover, the incidence and etiology of peritonitis episodes vary based on geographical region, and change over time [5].

However, to our knowledge, there were fewer studies about the risk factors associated with the first episode of peritonitis in Chinese PD patients. Therefore, we conducted this study, to get an improved understanding of the risk factors associated with the development of the first episode of peritonitis.

## Materials and Methods

### Patient Population

This single-center retrospective cohort study included all incident CAPD patients followed up in the PD center, The First Affiliated Hospital of Sun Yat-sen University, from 1 January 2006 to 31 December 2010. To satisfy the study protocol, all patients had to have received at least 3 consecutive months of PD therapy, and the exclusion criteria was the patients who were not maintained on PD more than 90 days, younger than 18 years old, or began PD in other centers and exchanged to follow up in our center. The dataset in the study are derived from included patient's files in our PD center. The study was conducted in compliance with the ethical principles of the Helsinki Declaration (http://www.wma.net/en/30publications/10policies/b3/index.html) and approved by the Human Ethics Committees of Sun Yat-sen University. Written informed consent was obtained from all participants.

The baseline characteristics of all patients within 1–3 months of the start of PD therapy were collected, including demographic data [age (years), gender, education level (senior middle school or above, junior high school or below), region where patients live(urban, rural), family incomes (thousand RMB/per year) (0 to 50, 50 and above), cause of end-stage renal disease (ESRD), previous hemodialysis, previous kidney transplant, major comorbidities (cardiovascular disease, such as myocardial infarction, angina, or history of congestive heart failure, cerebrovascular event, and peripheral vascular disease with or without amputation), hypertension, diabetes, malignancy, Charlson comorbidity index (CCI) score at the start of PD [6], body mass index (BMI) (kg/m^2^), type of catheter, operator], biochemical data [white blood cell (WBC), hemoglobin levels (g/l), hypersensitivity C-reactive protein(HsCRP) (mg/l), serum albumin levels (g/l), potassium (mmol/l), calcium(mmol/l), phosphate(mmol/l), calcium-phosphate product (Ca×P), blood urea nitrogen(BUN) (mmol/l), serum creatinine (umol/l),intact parathyroid hormone(iPTH) (pg/ml)], PD adequacy indices [renal and peritoneal Kt/V(urea clearance index) urea, residual urine volume(ml/24 h), baseline dialysate-to-plasma ratio of creatinine(D/Pcr)] and indices of nutrition [normalized protein catabolic rate (nPCR) (g/kg/d)], microbiological characteristics of the first episode of peritonitis, and total follow-up period (months). A standard peritoneal equilibration test was performed within the first 1–3 months after PD initiation. Patients were followed up until the primary end point, namely, the first episode of peritonitis. Those lost to follow-up were coded as censored observations, including death, transplantation, transfer to hemodialysis for reasons other than peritonitis and spontaneous recovery of renal function,or on 31 December 2012.

### Definition of Peritonitis and the First Episode of Peritonitis

Recorded episodes of peritonitis met at least 2 of the following 3 criteria from the 2010 International Society for Peritoneal Dialysis (ISPD) guideline recommendations [7]: 1. Symptoms of peritoneal inflammation such as abdominal pain; 2. An effluent white blood cell count exceeding 100/ml after a dwell time of at least 2 hours, with at least 50% polymorphonuclear neutrophilic cells; 3. Presence of organisms on Gram stain or subsequent culturing of PD fluid.

The first episode of peritonitis was defined as the first case in which cloudy peritoneal fluid was observed, with a leukocyte count equal to or greater than 100 cells/mm^3^, and with >50% polymorphonuclear cells.

Prescription of antibiotics was based on the patient’s clinical conditions according to the guideline of ISPD combined with our center’s experience. Patients were hospitalized whenever necessary.

### Statistical Analyses

Continuous data that are normally distributed are termed “parametric data,” and those that are not normally distributed, “nonparametric data.” Parametric data are presented as mean ± standard deviation. Nonparametric data are presented as median values with the interval from the 25th to the 75th percentile. Categorical variables are expressed as a percentage or ratio. Parametric data were compared by independent sample T-test or rank sum test. Nonparametric data were compared by the chi-square test. Demographic, socioeconomic, clinical data and PD adequacy variables were included in peritonitis risk analysis. Peritonitis-free survival curve was constructed according to Kaplan–Meier method. Univariate Cox proportional hazard regression was used to select variables associated with the first episode of peritonitis to the final multivariate Cox model and the elimination criterion for them was P>0.20. Collinearity among variables was tested and if statistically significant interactions were presented, one of them was excluded. All of the reported p-values are two-tailed, statistical significance was defined as P<0.05. All statistical analyses were performed using SPSS 18.0 (SPSS Inc.) software.

## Results

In a total of 1252 incident CAPD patients, 1117 patients 18 years or older who remained at least 90 days on PD were eligible for the study, whereas 62 were excluded for not completing 90 days on PD, 14 patients were younger than 18 years old and 59 patients began PD in other centers and exchanged to follow up in our center. The study population was followed up for a median of 26.1 months [interquartile range (IQR) 12.0–39.5 months]. Mean age at commencement of CAPD was 48.3±15.5 years (range 18–84 years). Elder (>65 years) and male patients comprised of a proportion of 16.5% and 58.6%, respectively. Etiologies of ESRD were chronic glomerulonephritis (58.7%), diabetic nephropathy (21.9%), hypertensive nephrosclerosis (6.2%), and other renal disease (13.2%).

**Figure 1:**
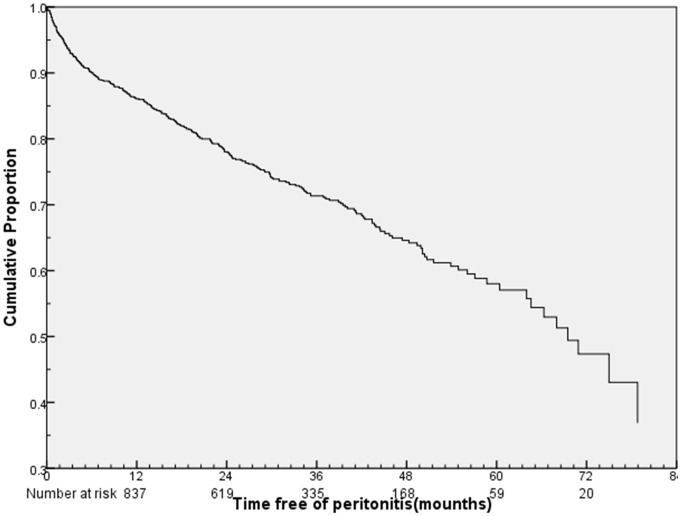
Peritonitis-free survival of 1117 incident peritoneal dialysis patients.

Three hundred and nine (27.7%) patients presented total 513 episodes of peritonitis during a cumulative follow-up period of 30,756.5 patient-months, whereas the remaining 808(72.3%) patients had no peritonitis. The overall peritonitis rate was 1/60 patient-months. The cumulative peritonitis-free survival was 86.2%, 78.1%, 71.4% and 57.8% at 1, 2, 3 and 5 year, respectively (Figure 1). Compared with the peritonitis-free patients, the patients with the first episode of peritonitis had advanced age, higher levels of CCI score, ALP, HsCRP (P<0.05) and lower levels of serum albumin (P<0.05) (Table 1).

**Table 1:** Baseline clinical characteristics and laboratory biochemistry data.

	Peritonitis-free group		first peritonitis group		P-value
	N	mean±SD, medain	N	mean±SD, medain	
Age(years)	808	47.1±15.5	309	51.8±14.9	**0.000**
Men (%)	475	58.8%	181	58.6%	0.738
Diabetes (%)	186	23.0%	67	21.7%	0.633
CCI score	808	3.51±1.81	309	4.10±2.21	**0.000**
BMI(kg/m^2^)	761	21.4±3.1	301	21.3±2.9	0.660
eGFR(ml/min1.73 m^2^)	592	3.71±2.96	222	3.64±2.62	0.756
Residual urine(ml/24 h)	596	736±489	231	723±510	0.732
D/P cr	460	0.71±0.12	172	0.71±0.12	0.957
Weekly Kt/V	592	2.39±0.68	223	2.36±0.67	0.553
nPCR(g/kg/d)	590	0.96±0.27	223	0.94±0.27	0.469
WBC(10^12^/L)	786	6.77±2.05	307	6.98±2.28	0.140
Hemoglobin(g/L)	786	103±20	306	101±22	0.157
Prealbumin(g/L)	554	337±101	195	322±102	0.080
AST(U/L)	765	23.7±14.2	299	25.0±17.1	0.233
ALT(U/L)	765	15(11, 23)	299	16(11, 23)	0.342
ALP(U/L)	764	77.3±35.3	298	88.6±61.2	**0.003**
Serum albumin(g/L)	768	37.8±5.1	299	36.4±5.5	**0.000**
HsCRP(mg/L)	545	1.60(0.58,5.93)	200	2.71(0.88, 9.17)	**0.002**
Sodium(mmol/L)	783	140±4	306	140±4	0.204
Potassium(mmol/L)	782	3.79±0.74	304	3.72±0.70	0.138
Calcium(mmol/L)	782	2.27±0.22	305	2.26±0.34	0.671
Phosphorus(mmol/L)	759	1.46±0.44	297	1.41±0.44	0.091
Glucose(mmol/L)	781	5.32±2.44	306	5.34±2.42	0.889
Total cholesterol(mmol/L)	702	5.10±1.33	283	5.09±1.31	0.949
HDL-C(mmol/L)	698	1.31±0.46	282	1.27±0.41	0.307
Urea nitrogen(mmol/L)	784	17.2±7.9	306	17.4±15.8	0.737
Creatinine (umol/L)	785	761±277	306	738±279	0.176
Uric acid(umol/L)	749	421±89	291	419±99	0.813
iPTH (pg/ml)	688	216(88, 408)	280	224(83,395)	0.942
Serum iron((umol/L)	596	8.20(5.70,12.0)	217	8.8(6.0,12.1)	0.220

CCI: Charlson comorbidity index; BMI: body mass index; eGFR: estimated glomerular filtration rate; D/P cr: dialysate-to-plasma ratio of creatinine; Kt/V: urea clearance index; nPCR: normalized protein catabolic rate; WBC: white blood cell; AST: Aspartate aminotransferase; ALT: Alanine aminotransferase; ALP: Alkaline phosphatase; HsCRP: high sensitivity C-reactive protein; HDL-C: High-density lipoprotein cholesterol; iPTH: intact parathyroid hormone.

Causative organisms of the first episode of peritonitis of the cohorts are summarized in following: 99 (32%) were due to gram-positive agents, 70 (22.7%) to gram-negative germs, and 18 (5.8%) to fungi. Among the gram-positive organisms, the most frequent was Streptococcus (11.7%), followed by Coagulase-negative staphylococci (11.3%), Staphylococcus aureus (4.2%) and Enterococcus (1.0%), the other gram-positive organisms (3.9%). Among the gram-negative organisms, Escherichia coli was the main gram-negative germ causing 12.9% of the cases, followed by Klebsiella pneumoniae (4.5%), Pseudomonas aeruginosa (0.3%), other gram-negative organisms(4.9%). Microbiological information was not reported in 42 (13.6%) episodes and negative culture was observed in 77 (24.9%). For the outcome of the first episode of peritonitis, 277 (89.6%) patients were cured, 22(7.1%) transferred to hemodialysis, 10 (3.2%) death, and the average time to death was 24.5 days (range: 3 to 53 days), and the outcome of the different organisms infection for the first episode of peritonitis in detail are showed in Table 2.

**Table 2:** Outcome of the different organisms infection for the first episode of peritonitis (n = 309).

Causative organisms	Sample size (n)	Cure (n, %)	Transfer to hemodialysis (n, %)	Death (n, %)	Average time to death (d)
Gram-positive organism	99	95 (96.0%)	2 (2.0%)	2 (2.0%)	25
Gram-negative organisms	70	66 (94.2%)	2 (2.9%)	2 (2.9%)	25
Fungi	18	1 (5.5%)	14 (77.8%)	3 (16.7%)	13
Multiple organisms	3	3 (100%)	0	0	0
Negative culture	77	72 (93.5%)	4 (5.2%)	1 (1.3%)	41
No microbiological information	42	40 (95.2%)	0	2 (4.8%)	32.5

The frequency of the first episode of peritonitis and the median time to the first episode of peritonitis according to demographic, socioeconomic, dialysis-related, clinical and PD adequacy variables are expressed in Tables 3 and 4. Age, educational level, and albumin were associated with risk for the first episode of peritonitis in univariate Cox analysis (Tables 3 and 4), and were entered in the multivariate Cox proportional hazard model. Gender, hemoglobin and potassium were also entered in the multivariate Cox proportional hazard model because their P value were <0.20 in univariate analysis. For the collinearity among variables, no significant interaction was found between variables.

**Table 3:** Frequency and median time to the first episode of peritonitis according to demographic factors**.**

Variable	N	First PeritonitisFrequency(%)	Median Time tothe First Episode ofPeritonitis (months)	P value
Age (years)				
≤65	934	250(26.8)	13.9	
>65	183	59(32.2)	10.2	**0.009**
Gender				
Women	461	128(27.8)	18.3	
Men	656	181(27.6)	10.2	0.166
Educational level				
≥Senior middle school	514	126(24.5)	13.0	
≤Junior high school	533	169(31.7)	15.7	**0.017**
Income (thousand RMB/per year)				
≤50	786	218(27.7)	16.0	
>50	162	43(26.5)	6.2	0.631
Region where patients live				
Urban	713	203(28.5)	14.2	
Rural	393	102(26.0)	10.9	0.865
Catheter type, tip				
Straight	961	263(27.4)	11.8	
Coiled	149	44(29.5)	18.2	0.279
Previous hemodialysis				
Yes	39	9(23.1)	11.0	
No	1078	300(27.8)	13.4	0.593
Previous Kidney transplantation				
Yes	9	3(33.3)	14.6	
No	1108	306(27.6)	13.2	0.971
Operator				
Self	1045	290(27.8)	13.8	
Else	71	18(25.4)	5.9	0.527

**Table 4:** Frequency and median time to the first episode of peritonitis according to clinical factors.

Variable	N	First PeritonitisFrequency (%)	Median Time to theFirst Episode ofPeritonitis (months)	P value
Diabetes				
No	864	242(28.0)	13.0	
Yes	253	67(26.5)	15.3	0.959
Cardiovascular disease				
No	819	222(27.1)	13.4	
Yes	298	87(29.2)	13.1	0.357
Hypertension				
No	797	216(27.1)	13.4	
Yes	320	93(29.1)	13.1	0.374
Malignancy				
No	1092	301(27.6)	13.4	
Yes	25	8(32.0)	7.0	0.406
BMI(kg/m^2^)				
<18.5	177	52(29.4)	22.5	0.962
18.5–24.9	761	215(28.3)	11.6	
25–29.9	110	32(29.1)	8.0	0.704
≥30	14	2(14.3)	12.7	0.341
Ca×P				
<55	930	260(28.0)	14.0	
≥55	143	42(29.4)	5.9	0.827
Hemoglobin(g/l)				
≥110	400	100(25.0)	15.9	
<110	679	194(28.6)	13.1	0.081
Potassium (mmol/L)				
≥3.5	686	177(25.8)	11.8	
<3.5	400	127(31.8)	16.0	0.123
Serum albumin(g/L)				
≥38	567	139(24.5)	13.4	
<38	500	160(32.0)	13.8	**0.000**
Residual urine				
≥100	797	221(27.7)	15.3	
<100	31	11(35.5)	14.6	0.201
nPCR(g/kg/d)				
≥0.8	581	158(27.2)	16.1	
<0.8	232	65(28.0)	11.1	0.632
Weekly Kt/V				
≥1.7	720	201(27.9)	14.2	
<1.7	95	22(23.2)	23.7	0.653

The multivariate analysis by using independent variables as categorical data demonstrated that factors independently associated with increased hazard risk (HR) for the first episode of peritonitis were old age(>65 years versus ≤65 years: HR = 1.427, 95% CI = 1.051 to 1.938, P = 0.023), male(male versus female: HR = 1.315, 95% CI = 1.028 to 1.684, P = 0.030), lower education level (≤ junior high school versus ≥senior middle school: HR = 1.446, 95% CI: 1.127 to 1.855, P = 0.004) and serum albumin (<38 g/L versus ≥38 g/L: HR = 1.425, 95% CI: 1.112 to 1.825, P = 0.005) (Table 5). The result by using independent variables as continuous data showed that factors associated with risk for the first episode of peritonitis were advanced age (HR = 1.016, 95% CI = 1.007 to 1.024, P = 0.000), male (HR = 1.376, 95% CI = 1.079 to 1.756, P = 0.010) and lower albumin level (HR = 0.961, 95% CI = 0.937 to 0.985, P = 0.002), which was similar with the results analyzed with categorical data.

**Table 5:** Cox proportional hazards regression model.

Variable	HR	95% CI		P value
		Lower	Upper	
Age >65 years	1.427	1.051	1.938	**0.023**
Gender: male	1.315	1.028	1.684	**0.030**
Educational level ≤ junior high school	1.446	1.127	1.855	**0.004**
Serum albumin <38 g/L	1.425	1.112	1.825	**0.005**
Hemoglobin <110 g/L	1.184	0.916	1.529	0.198
Potassium <3.5 mmol/L	1.154	0.906	1.470	0.245

Cox proportional hazards regression model (n = 309 episodes in 1117 patients).

## Discussion

Our retrospective cohort study showed that the proportion of patients with the first episode of peritonitis was 27.7%. The cumulative peritonitis-free survival was 86.2%, 78.1%, 71.4% and 57.8% at 1, 2, 3 and 5 year, respectively. Older age, male, lower educational level and hypoalbuminemia were associated with the first episode of peritonitis.

Our result identified that the elderly patients (>65 years) was a risk factor for the first episode of peritonitis. The result was in accordance with the ANZDATA [4] analysis, which identified Aboriginal race, obesity, and older age as predictors of peritonitis. On the contrary, the USRDS results [8] and a multicenter observational study in BRAZPD [9] showed that older age was not a predictor of peritonitis risk. Recently, a single-center study manifested elderly patients were at higher risk of peritonitis episodes [10]. This finding could be explained by the decreased dexterity and vision in elderly patients, which can be a hindrance to proper aseptic technique. Moreover, it can be explained by the association between the older age and comorbidities [3].

Several studies have observed a higher rate of peritonitis in women, and most caused by gram-negative bacilli derived from vaginal contamination [11]. However, an interesting finding of the present study was that men were associated with the high risk for the first episode of peritonitis as compared to women. So the reason for our result may be as follow: firstly, this maybe because the obedience of men was not better than women; Secondly, it may due to the more social interaction needs for men than women, and some men even still insist on working during the dialysis interphase, which may cause the fluctuations of patient's living environment and thus may be impacted by the poor environmental in surrounding. One point that what we should not ignore was women maybe more careful and had better personal hygiene condition than men in the daily life. Last but not the least, it is possible that men comprised 58.7% of our study group.

Regarding educational level, Martin *et al*. [9] reported that the influence of educational level on peritonitis risk was observed after adjustment for the socioeconomic and demographic characteristics as well as relevant coexisting medical factors and comorbidities, showing the strong influence of educational level on peritonitis risk. A similar result reported by Chern *et al* [12], showed that a lower education level was a major risk factor for peritonitis, independent of age, sex, hypoalbuminemia, and comorbidities. We also found an association between lower education level and the first episode of peritonitis which was in accordance with these reports. Lower education level maybe associated with the decreased ability to learn the operation procedure and associated knowledge about CAPD, so to build a comprehensive and long-time CAPD education program according to the patients’ different educational level is more important, especially for CAPD patients with lower education level.

Hypoalbuminemia in baseline was a strong risk factor for the first episode of peritonitis in the present study. The influence of hypoalbuminemia on peritonitis risk has been previously reported. Studies in the United States [13] and Hong Kong [14] found that every 1 g/dL decrease of serum albumin concentration increased the risk of peritonitis by 74% and 67% respectively. In our study, compared with the patients with a baseline serum albumin level of 38 g/L or greater, patients with a baseline serum albumin level less than 38 g/L had a hazard ratio of 1.406(HR = 1.406) for the first episode of peritonitis. It was well known that hypoalbuminemia is the result of malnutrition and inflammatory response and may lead to susceptibility to infection. Therefore, the intervention measures should be given to improve of patients’ nutritional status to prevent malnutrition in CAPD patients.

Actually, in contrast to the previous studies, diabetes, socioeconomic status, transferring from HD to PD, and BMI did not influence the first episode of peritonitis in our study. Data on diabetes have been conflicting, but the majority of authors have reported that presence of diabetes is associated with higher peritonitis risk. For our center, diabetes was not the major cause of ESRD, and the technique survival in CAPD patients with diabetes was similar to those without diabetes [15]. Regarding socioeconomic status, Chow *et al*. [16] showed that the need for social security assistance and illiteracy were the only statistically significant factors associated with time to the first episode of peritonitis, after adjustment for age and medical factors, such as diabetes and serum albumin. However, Andrade, B. K *et al*. [17] reported that economic status is not independently associated with survival in a large cohort and should not be considered a barrier for PD indication, and our result was in line with the latter.

In this study there was a high proportion of culture-negative episodes. The antibiotic drugs used in the local hospital before they transferred to our hospital may resulted in higher culture-negative rate. In addition, we should review the laboratory methods currently utilized and generate new protocols to improve the results in our center.

The main strength of this study is the fact that it was a large single-center cohort study of incident CAPD patients, incorporating several potentially important demographic, socioeconomic, PD adequacy, residual renal function, and educational factors. Moreover, this is the first Southern China study designed to address whether these factors are predictors for the first episode of peritonitis risk. However, our study has several limitations. First, this single-center study was based on retrospective data and the data of some induces was missing, and this may lead to the biased results. Moreover, our data set did not include the data on dedicated room to dialysis operate, exit site catheter care, whether wearing a face mask, patient's hand hygiene condition and patients’ compliance, all the factors that could represent peritonitis risk factors. In addition, it has been reported [18] that fluid excess associated with protein-energy wasting, old age, and decreased residual urine output. But the relationship between the volume status and peritonitis is worth for further study. Hence, further studies with more complete data collection and multicenter involvement will be needed to identify the factors associated with PD-related peritonitis in Southern China.

## Conclusions

Older age, male, lower educational level and hypoalbuminemia at initiation of PD were related to the first episode of peritonitis, independent of hemoglobin and potassium. These results may be helpful in identifying the patients starting PD treatment who are at the highest risk for the first episode of peritonitis, and could help to take intervention to prevent and improve CAPD outcomes.
